# The Essential Thrombocythemia, Thrombotic Risk Stratification, and Cardiovascular Risk Factors

**DOI:** 10.1155/2020/9124821

**Published:** 2020-03-27

**Authors:** Salvatrice Mancuso, Vincenzo Accurso, Marco Santoro, Simona Raso, Angelo Davide Contrino, Alessandro Perez, Florinda Di Piazza, Ada Maria Florena, Antonio Russo, Sergio Siragusa

**Affiliations:** ^1^Hematology Division University Hospital Policlinico “Paolo Giaccone”, Via del Vespro 129, 90127 Palermo, Italy; ^2^Department of Surgical, Oncological and Stomatological Disciplines, University of Palermo, Via del Vespro 129, 90127 Palermo, Italy; ^3^Department of Surgical, Oncological and Stomatological Disciplines, Section of Medical Oncology, University of Palermo, Via del Vespro 129, 90127 Palermo, Italy; ^4^Department of Sciences for Promotion of Health and Mother and Child Care, Anatomic Pathology, University of Palermo, Palermo, Italy

## Abstract

Essential thrombocythemia is a rare hematological malignancy with good overall survival, but moderate to high risk of developing arterial or venous thrombosis lifelong. Different thrombotic risk scores for patients with essential thrombocythemia have been proposed, but only one of them (the IPSET-t scoring system) takes into account the classical cardiovascular risk factors as one of the scoring items. Currently, in clinical practice, the presence of cardiovascular risk factors in patients with diagnosis of ET rarely determines the decision to initiate cytoreductive therapies. In our study, we compared different risk models to estimate the thrombotic risk of 233 ET patients and the role of specific driver mutations and evaluated the impact that conventional cardiovascular risk factors (hypertension, cigarette smoking, diabetes, obesity, and dyslipidaemia) have on thrombotic risk in patients with ET. Perspective studies conducted on a polycentric large cohort of patients should be conducted to estimate the impact of cardiovascular risk factors in determining thrombosis in ET patients, evaluating the opportunity of initiating a cytoreductive therapy in patients with cardiovascular risk factors, even if classified into low to moderate risk groups according to other scoring systems.

## 1. Introduction

Essential thrombocythemia is a rare hematological malignancy characterized by elevated platelet count in peripheral blood and increased megakaryocytes proliferation in bone marrow, whose diagnosis criteria were recently revised in the 2016 World Health Organization revised classification of myeloid malignancies [1]. The estimated ET annual incidence is estimated at 0.6–2.5 cases per 100,000 [2]. Interestingly, ET is characterized by overall favourable prognosis if compared to the other myeloproliferative neoplasms (MPNs) (although life expectancy in ET is inferior to the control population) [3], increased risk of thrombohemorrhagic complications, and possible evolution in myelofibrosis and acute leukemia.

In a recently published study performed on a cohort of 826 Mayo Clinic patients with ET, PV, or PMF, the median survivals were approximately 20 years for ET, 14 years for PV, and 6 years for primary myelofibrosis [4]. An increased risk of vascular complications over time is the main clinical feature of ET. In a study conducted on 1297 patients, thrombotic events, before or at the time of ET diagnosis, were reported in 231 cases (17.8%) [5]. The conventional thrombotic risk stratification in ET distinguishes patients into two risk groups: a high-risk group includes patients older than 60 years or with history of thrombosis, whereas the absence of both risk factors identifies the low-risk group [6]. The thrombotic risk score named IPSET-t takes into account the cardiovascular risk factors (CVR) and stratifies patients into low-, intermediate-, and high-risk group [7]. Recently, a revision of IPSET-thrombosis was achieved by the re-analysis of the original IPSET-thrombosis data set. The revised IPSET-thrombosis (r-IPSET-t) uses three adverse variables to delineate four risk categories, excluding CVR form the risk determinants [8]. In 2017, a further enhancement of the IPSET-t was proposed by Tefferi and Barbui adding the negative effect of MPL mutation [9]. In an article entitled *“Comparison between thrombotic risk scores in essential thrombocythemia and survival implications*,*”* we used the three available risk scores to classify the same monocentric group of patients, highlighting how many patients change thrombotic risk class when reclassified, although the high-risk group still remains the larger [10]. Today, the classification of thrombotic risk according to r-IPSET-t in patients with ET do not take into account CVR, and these conditions do not currently influence the choice of cytoreductive therapy in clinical practice, even if it could suggest to adopt a different dosage of acetylsalicylic acid or other antiplatelet drugs for thrombosis prophylaxis [11–14]. In this report, we compared different risk models in order to estimate the thrombotic risk in a cohort of 233 patients with ET. Moreover, we evaluated the frequency of CVR conditions in our ET patients and its possible impact on the thrombotic risk.  Table 1 reports the different thrombotic scores and how they are calculated.

## 2. Methods

We applied the three different thrombotic risk models available in clinical practice (the conventional model, the IPSET-t model, and the r-IPSET-t) to a large group of 233 ET patients followed up in our hematology unit, over a period of 23 years (from 1997 to 2018). Inclusion criteria were diagnosis of ET according to WHO criteria. Criteria were revised according to 2016 WHO revision for the oldest diagnosis dates. The data collection, filing, and use were made in accordance with the Helsinki Declaration. The Local Ethics Committee approved the data collection and use in relation to the aim of the research. Patients were treated with antiplatelet prophylaxis and cytoreductive therapy as per clinical practice, according to guidelines. The IPSET-t score contemplates the assignment of 2 points each for previous thrombotic event or for the presence of JAK2-V617F mutation, 1 point for age greater than 60 years, and 1 point for the presence of ≥1 CVR. The low risk is defined by a score lower than 2, the intermediate risk by a score equal to 2, and the high risk by a score greater than 2. The r-IPSET-t uses three adverse variables to identify 4 risk groups: age > 60, thrombosis history, and JAK2-V617F presence. Indeed, patients younger than 60 years old, negative history of thrombosis, and no JAK2-V617F mutation are considered at very low risk; patients with JAK2-V617F mutation but no thrombosis history are considered at low risk; patients with JAK2-V617F mutation with a diagnosis of thrombosis are considered at intermediate risk; high-risk category is defined by the contemporary presence of the three risk factors.

We performed a comparison between the traditional assessment system for the thrombotic risk and the IPSET-t prognostic score as well as a comparison between the IPSET-t prognostic score and the r-IPSET-t stratification system. We evaluated the main characteristics of the study population at diagnosis such as gender, age, and mutational status along with CVR frequency, such as cigarette smoking habits, hypertension, diabetes, obesity, and dyslipidaemia. In particular, patients with only one of these conditions were distinguished by those with more than one cardiovascular risk factor or without CVR. Moreover, the relation between these cardiovascular risk conditions and the onset of thrombosis has been evaluated. The frequencies were calculated using the chi-square method, and the comparison between medians was performed through the Kruskal-Wallis test.

## 3. Results

**Figure 1:**
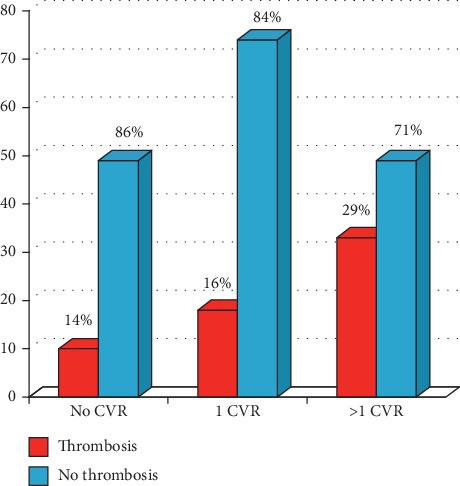
Thrombosis and cardiovascular risk factors in 233 ET patients. The percentages refer to portions of each subgroup identified by number of CVR (no CVR, only 1 CVR, and more than 1 CVR).

The clinical and laboratory characteristics, the frequencies of each cardiovascular risk factor of the cohort under examination are respectively summarized in Tables 2 and 3. Respecting to the traditional system, IPSET-t prognostic score identifies the intermediate-risk category within which can be placed patients dispersed in the low- and high-risk category according to the previous classification (see Table 4). Therefore, of 68 “traditionally” low-risk patients, only 32 patients (47%) were reclassified as low-risk using the IPSET-t model, while 17 patients (25%) were newly classified as intermediate risk and 19 (27.9%) as high risk. The “traditional” high-risk category, instead, was almost conserved, when patients were reclassified using IPSET-t (87.9% of the traditionally high-risk patients fell into the high-risk category according to the IPSET-t). Furthermore, by applying the r-IPSET-t stratification to our group of patients, low-risk patients, according to IPSET-t, were almost completely redistributed between very low risk and intermediate-risk categories, while IPSET-t intermediate-risk patients fell into the low-risk group. On the contrary, the IPSET-t high-risk category was the most conserved, with 86% of the patients classified in the same risk category when using the r-IPSET-t (Table 5). In Table 6, the JAK2/CALR/MPL mutational status of 61 patients with arterial or venous thrombosis has been listed. In particular, the genetic variant frequency is 80.4% (*n* = 49), 1.6% (*n* = 1), 1.6% (*n* = 1), and 16.4% (*n* = 10) for JAK2, CALR, MPL, and triple negative, respectively. Moreover, within our group of patients, 61 ET patients had thrombosis (50 arterial and 11 venous); of 61 cases, 48 (20.6%) presented a thrombosis at or before diagnosis and 14 (6.01%) after diagnosis. In our patients with ET, the frequency of thrombotic episodes is strictly correlated with the presence of CVR. In fact, the overall thrombosis frequency is lower in patients without CVR (10/59) if compared to patients with only one cardiovascular risk factor (18/92) or to patients with more than one cardiovascular risk factor (33/82) (Figure 1).

## 4. Discussion

The recent thrombotic risk stratification for ET patients allows a more detailed and feasible patients assessment. Interestingly, either using the traditional system or the IPSET-t prognostic score as well as the r-IPSET-t for the assessment of the thrombotic risk, high-risk patients are always the most represented (70.8%, 70.3%, and 60.9%, respectively). This evidence is of note, being the high-risk category always indicated for cytoreduction that can affect the patient's quality of life, despite the good overall prognosis of patients with ETin general. Moreover, we confirmed that CALR mutations confer a lower thrombotic risk if compared to JAK2 mutations and to a triple negative mutational status. In ET, thrombotic complications and cardiovascular events are very frequent. Therefore, the evaluation of the thrombotic risk in this clinical setting is crucial to choose the proper therapeutic approach. Indeed, the new classification for the thrombotic risk in ET identifies 4 categories: very-low, low, intermediate, and high (see Introduction). Unfortunately, cardiovascular risk factors are not yet considered. In a previously proposed thrombotic risk classification model, at the traditional high- and low-risk category was added an intermediate-risk category specific for all the patients aged under 60 years, with no history of thrombosis but with the presence of cardiovascular risk factors [12]. This thrombotic risk classification model is generally not followed.

Cerquozzi et al. explored the association between CVR and the occurrence of arterial or venous events at or following diagnosis; they found that older age (≥60 years), hypertension, diabetes, hyperlipidemia, and normal karyotype were associated with arterial events, whereas younger age (<60 years), female sex, palpable splenomegaly, and history of major hemorrhage were associated with venous events [13]. In a study by Lekovic et al., risk scores were assigned according to multivariable analysis–derived hazard ratios (HR) for the presence of 1 CV risk factor (HR = 3.5; 1 point), >1 CV risk factors (HR = 8.3; 2 points), and previous thrombosis (HR = 2.0; 1 point). A final three-tiered prognostic model for thrombosis risk prediction was developed as low (score 0), intermediate (score 1 or 2), and high (score 3). The hazard of thrombosis was 3.8% in the low-risk group, 16.7% in the intermediate-risk group, and 60% in the high-risk group (*p* < 0.001) [14]. Today for patients with PV or ET with less than 60 years and with one or more cardiovascular risk factors, there is no indication for cytoreductive therapy. Only for ET, the IPSET-thrombosis system that includes age, previous thrombosis, cardiovascular risk factors, and JAK2-V617F mutation is the recommended prognostic system and it should be scored in all patients at diagnosis. This implies that general risk factors for thrombosis, including smoking habits, diabetes mellitus, arterial hypertension, and hypercholesterolemia, should also be considered even if the absence of specific therapeutic indications still remains [15]. Our study suggests the importance of CVR in determining the thrombotic risk stratification in ET patients and demonstrates how the frequency of thrombotic events increases along with the number of CVR.

The retrospective nature and the long interval of time in which the patients were diagnosed and followed up are weak points of our work. Perspective studies should be undertaken to assess the role of CVR in determining thrombotic events in ET. These studies should also evaluate the opportunity to perform cytoreductive therapy in patients with ET aged less than 60 years, but with one or more CVR.

## Figures and Tables

**Table 1:** The traditional system for thrombosis risk in ET, the IPSET-t, and the revised IPSET-t.

Traditional score for thrombotic risk in ET				
Low risk	Age <60 years	AND	No thrombosis	
High risk	Age >60 years	OR	Thrombosis	

IPSET-thrombosis score				
Items	Points	Low risk	Intermediate risk	High risk
JAK2 V617F	2	<2 points	2 points	>2 points
Thrombosis	2			
Age >60 years	1			
CVR ≥1	1			

Revised IPSET-thrombosis				
Very low risk	Age <60 years	AND	No thrombosis	JAK2 WT
Low risk	Age <60 years	AND	No thrombosis	JAK2 V617F
Intermediate risk	Age >60 years	AND	No thrombosis	JAK2 wildtype
High risk	Age >60 years	OR	Thrombosis	JAK2 WT/V617F

CVR: cardiovascular risk factors; WT: wildtype.

**Table 2:** Clinical and laboratory characteristics of the ET patients included in the study.

Patients characteristics	
Number of patients	233
Median follow-up (months)	48.4 (range 0.3–313)
Median age at diagnosis (years)	65.9 (range 14.5–92)
Age <60 years	87 (37.3%)
Age >60 years	146 (62.7%)
Female	160 (68.7%)
Male	73 (31.3%)
1 CVR	92 (39.5%)
>1 CVR	82 (35.2%)
JAK2 V617F positive/tested	168/233 (72.1% of the total number of pts)
CALR mutated/tested	21/65 (9% of the total number of pts)
MPL mutated/tested	4/44 (1.7% of the total number of pts)
Triple negative	40 (17.2% of the total number of pts)
Thrombosis before or at diagnosis	48 (20.6%)
Thrombosis after diagnosis	14 (6%)

**Table 3:** Cardiovascular risk factors in the 233 ET patients included in the study.

Cardiovascular risk factors	#	%
Hypertension	148	63.5
Dyslipidaemia	56	24
Diabetes	33	14.2
Cigarette smoke exp.	31	13.3
Obesity	21	9

**Table 4:** Distribution of the 233 ET patients according to the thrombotic risk calculated with the traditional system and the IPSET-t score.

IPSET-t score	r-IPSET-t score				
	Very low	Low	Intermediate	High	Total
Low risk	26 (66.7%)	3 (7.7%)	10 (25.6%)	0	39
Intermediate risk	0	17 (56.7%)	11 (36.7%)	2 (6.7%)	30
High risk	0	14 (8.5%)	9 (5.5%)	141 (86%)	164
Total	26	34	30	143	233

**Table 5:** Distribution of the 233 ET patients according to the thrombotic risk calculated with the IPSET-t score and the r-IPSET-t score.

Traditional system	IPSET-t score			
	LOW	Intermediate	High	Total
Low risk	32 (47%)	17 (25%)	19 (28%)	68 (100%)
High risk	7 (4.3%)	13 (0.8%)	145 (87.9%)	165 (100%)
Total	39	30	164	233

**Table 6:** JAK2, CALR, and MPL mutational status in the 61 ET patients with thrombosis.

Mutation revealed in ET pts with thrombosis	#	%
JAK2 V617F	49	80.4
CALR	1	1.6
MPL	1	1.6
Triple negativity	10	16.4
Total	61	100

## Data Availability

The data used to support the findings of this study are available from the corresponding author upon request.
